# Supplementary material for: Adjusting for principal components can induce spurious associations in genome-wide association studies in admixed populations
Source: bioRxiv. 2024 Apr 3:2024.04.02.587682. Preprint. [Version 1] doi: 10.1101/2024.04.02.587682 (PMC11014513; doi:10.1101/2024.04.02.587682)
Supplement: Supplement 1 [file NIHPP2024.04.02.587682v1-supplement-1.pdf]

bioRxiv preprint doi: https://doi.org/10.1101/2024.04.02.587682; this version posted April 3, 2024. The copyright holder for this preprint (which was not certified by peer review) is the author/funder, who has granted bioRxiv a license to display the preprint in perpetuity. It is made available under aCC-BY 4.0 International license.

Supporting Information

S1 Text. Supplemental Information. Includes 15 figures, proofs and simulations validating the theoretical results presented in the main paper, and a list of WHI investigators.
